# Effectiveness of social video platforms in promoting smoking cessation among youth: A content-specific analysis of smoking cessation topic videos on the social platform Bilibili

**DOI:** 10.18332/tid/169662

**Published:** 2023-08-21

**Authors:** Liujiang Ye, Yijie Ye, Hao Gao

**Affiliations:** 1 School of Journalism and Communication, Nanjing Normal University, Nanjing, China

**Keywords:** smoking cessation, social media, Bilibili, health promotion

## Abstract

**INTRODUCTION:**

Smoking cessation is a significant public health issue for young people. Social media provide the public with health knowledge through various types of videos. Bilibili is a trendy social video platform among the young population in China, and the number of smoking cessation videos on this platform is continuously increasing. Different content creators advocating smoking cessation through videos may influence young people’s attitudes and behaviors towards tobacco and smoking. This study aims to measure the message sensation value (MSV) and the information appeals in smoking cessation videos on Bilibili, examining their impact on communication effectiveness.

**METHODS:**

This study collected 337 videos from Bilibili and conducted a content analysis regarding the content creator’s information, video presentation, MSV, and message appeals. The communication effectiveness of the videos was defined as a dependent variable and was divided into three dimensions: communication breadth, recognition, and participation.

**RESULTS:**

The average MSV (rated on a scale of 0 to 11) for smoking cessation-themed videos was 4.49 (SD=2.23). Chi-squared analysis revealed significant differences among different types of videos in the use of threat appeal (p<0.001), humor appeal (p<0.001), and psychological benefit (p<0.05). Additionally, different types of creators showed differences in the use of threat appeal (p<0.05), humor appeal (p<0.001), and psychological benefit (p<0.05). ANOVA results indicated significant differences in the level of MSV among different smoking cessation videos (F=39.775, p<0.001). Linear regression analysis showed that MSV, threat appeal, humor appeal, and economic benefit positively impacted dissemination effects (p<0.001).

**CONCLUSIONS:**

The results indicate that young people are likelier to watch smoking cessation videos with higher MSV and information appeal. These elements can enhance persuasion and the effectiveness of communication. Therefore, when video creators aim to promote smoking cessation among young people, they can consider factors such as MSV, threat appeal, humor appeal, and economic benefit to enhance communication effects.

## INTRODUCTION

Tobacco use is a major cause of non-communicable diseases and death, with over 8 million people dying yearly from smoking-related causes^1^. Numerous studies consistently indicate that tobacco use is a significant risk factor associated with cancer^2^. Smoking and mental health problems are highly comorbid among young people^3^. Therefore, effective tobacco control is urgently needed.

Social media have been crucial in promoting health and educating about various diseases^4^. Social media can provide services for young adult smokers seeking smoking cessation help^5^. Research shows that interactive web-based smoking cessation interventions contribute to quitting smoking^6^. Since young people are the primary social media users, targeting smoking cessation promotion at young people through social media is considered a practical approach.

Among various social media platforms, online videos have become the preferred form of content consumption for people, especially young individuals. As of June 2022, China has seen significant growth in the user base of online videos, reaching 962 million users^7,8^. Bilibili, as one of the video platforms, has the youngest user base among Chinese internet users, with 90% being users aged <25 years and often associated with youth culture^9,10^. Previous studies have shown that Bilibili also plays a positive role in promoting health among young people^11^. Therefore, it is worth exploring whether Bilibili actively promotes smoking cessation.

The success of health promotion through the media often depends on the persuasiveness and impact of the transmitted information^12^. When measuring the effectiveness of video dissemination, studies have shown that both MSV (message sensation value) and message appeal are crucial indicators for assessing the persuasive strength of video information^13^. MSV refers to the extent to which the audio-visual characteristics of television messages evoke sensory, emotional, and arousal responses^14^. MSV captures their attention to the information by inducing emotional reactions in respondents, leading to persuasion^15^. A higher MSV results in a more significant persuasive impact^16^. Research has demonstrated the effectiveness of high MSV in antismoking advertisements targeting young people^17^.

Message appeal refers to the information used to attract consumers’ attention and influence their perception of the product^15^. Different types of message appeals, whether rational or emotional, can influence the persuasive effects of information recipients^18^. In smoking cessation research, the three most prominent message appeals are threat, social, and humor appeal^19^. Additionally, research indicates that economic and psychological effects also play a role in reducing harm from electronic cigarettes in videos^20^.

Therefore, this study aims to explore the influence of MSV and message appeal, as two indicators for evaluating information persuasiveness, on smoking cessation videos on Bilibili. It aims to provide specific strategies for social media, especially social video platforms, to enhance the communication effectiveness of smoking cessation promotion.

## METHODS

### Sample

Using the keyword ‘smoking cessation’, the research team searched for smoking cessation-themed videos on Bilibili in 2022, resulting in 423 videos. The research team manually filtered the data to exclude videos unrelated to smoking cessation or that did not meet the coding criteria. Ultimately, 337 videos were selected as the study sample. These videos contained information such as URLs, publishers, view counts, share counts, comment counts, like counts, danmaku (bullet comment) counts, and coin counts (a Bilibili feature where fans can reward video creators).

The sample data in this study exhibited a generally normal distribution. To further confirm the normality, the Kolmogorov-Smirnov test was employed with a significance level of p<0.05, considering the skewness and kurtosis values as references. The criteria set were absolute skewness values less than three and absolute kurtosis values less than 10, indicating a normal distribution^21^. For data that did not follow a normal distribution, appropriate transformations, such as logarithmic transformations, were applied to address the issue.

### Coding

*Dependent variable*

Following the approach proposed by Chen et al.^19^, the communication effects of the videos were categorized into four dimensions: overall communication effect, communication breadth, communication recognition, and communication participation. The overall communication effect was measured using the logarithmic sum of seven indicators: shares, views, likes, favorites, coins, danmaku (bullet comment), and video comments^11^. The number of shares^19^ measured communication breadth. Communication recognition was assessed using the logarithmic sum of four indicators: views, likes, favorites, and coins^19^. The number of danmaku and comments^19^ measured communication participation.

*Independent variable*

This study referred to the Lang et al.^22^ definition of MSV and the category design in the MSV coding scheme by Paek et al.^13^. Additionally, considering the characteristics of the sample and referring to the category construction by Palmgreen et al.^23^, several variables were modified or removed during the development of the coding scheme. For instance, the category ‘Loud/Fast Music’ was removed from the audio type category. After preliminary observations of smoking cessation video samples on Bilibili, it was determined that elements such as evidence and norms in the persuasion message features played a more significant role in persuasion. As a result, the coding scheme by Paek et al.^24^ was adjusted, replacing ‘Acted out’ and ‘Unexpected format’ with ‘Norms’ and ‘Evidence’, respectively. The MSV dimension coding comprised three types and twelve items (Table 1). In addition to using threat, social, and humor appeals, we also included psychological and economic benefits in the coding of message appeals (Table 1). During coding, a binary approach was used to determine whether the five types of appeals were present in the samples, with 0 indicating the absence and 1 indicating the presence of each appeal^25^.

**Table 1:** Operational definitions of variables

*Variables*	*Perreault and Leigh Index*
**Types of creators**	0.98
1) Individuals 2) Medical workers, 3) Media, 4) Corporate companies	
**Video type**	0.76
1) Vlog, 2) Showcase, 3) Animation, 4) Acting, 5) Documentary, 6) News, 7) Mashup video, 8) Oral speech, 9) Pictorial slideshow, 10) Interview	
**Video length**	0.93
1) <30 s, 2) 30–59 s, 3) 1 min –1 min 59 s, 4) 2 min – 2 min 59 s, 5) 3 min – 4 min 59 s, 6) ≥5 min	
**Message appeals**	
Threat appeal: the overall impression is that you will suffer in some way if you smoke by showing cancer patients, gross teeth or lungs, and scary images of people who suffer from smoking-related diseases.	0.88
Social appeal: visuals and major headlines convey that you will have more friends, dates, and popularity if you do not smoke.	1
Humor appeal: play on words, puns, or nonsensical statements.	0.88
Psychological benefit: Visuals and major headlines may move beyond outward appearance to convey smoking cessation’s impact on self-esteem and self-confidence.	0.93
Economic benefit: Overall, you will achieve career success and financial security or benefit from smoking cessation.	0.93
**Message sensation value**	
**Video/images**	
Number of cuts: the number of times the camera cuts from one visual scene to the next. Converted to low (0–6), moderate (7–14), and high (≥15) levels and coded as 0, 1, or 2.	0.83
Visual effects: anything beyond the range of human ability involving special visual effects.	0.98
Slow motion: the slowing of real-life action through technical intervention.	0.98
Bold or unusual colors: unusual colors outside the range of colors usually perceived in real life.	1
Intense images: intense or horrifying images including needles going into arms, guns pointed at heads or death.	0.85
**Audio/music**	
Sound saturation: background sound throughout the video clip, including street noise or other sounds, rather than simply a person talking throughout the video clip.	0.76
Background music: music to accompany the dialogue or action of the video clip.	0.98
Sound effects: unusual sounds (those that could not have occurred in real life) heard in the video clip, including gongs and other noises.	0.93
**Content**	
Acted out: instead of being told about the dangers of smoking, viewers see actions corresponding to the point of the smoking cessation video clip.	0.88
Surprise/twist ending: the presence of a climactic, shocking end to the smoking cessation video clip.	0.83
Evidence: The use of evidence enhances the credibility of the message source.	0.8
Norms: Messages can specify what ought to be done or avoided (injunctive norms) or what is typical (descriptive norms).	0.83

Referring to the coding scheme by Stellefson et al.^26^, the citation of video creator types was incorporated as one of the coding indicators. Video creators were classified into four types: individuals, healthcare professionals, media, and companies (Table 1). We classified the video types by drawing on references such as Niederdeppe^17^ and Li et al.^27^ and considering the characteristics of the sample videos (Table 1)^17,27^.

*Reliability test*

Two coders jointly performed the coding for this study. Before the official coding process, the coders randomly selected 10% of the video samples for pre-coding and conducted a HOLSTI reliability test to assess the agreement between the coders^28^. After two training rounds, the inter-coder reliability coefficient between the two coders was 0.901, indicating good reliability.

### Statistical analysis

Nominal variables were presented as frequency (%), and the chi-squared test was used to examine the associations between creator and video type, MSV, and message appeal. ANOVA was employed to analyze the relationships among creator types, video types, and MSV. In cases of missing values, the mode was used to fill in nominal variables, while the mean was used for quantitative variables. Linear regression analysis examined the relationships between the independent and dependent variables (communication effect). IBM SPSS Statistics 26.0 was utilized to process all coded data, and the statistical significance level was set at p<0.05 for all two-tailed tests.

## RESULTS

### Overview of videos related to smoking cessation

Among the 337 videos, individual users accounted for the highest proportion, approximately 48.1%, followed by media at 35.4%. Healthcare professionals were relatively fewer, accounting for 10.3%, while corporate creators were the least represented, comprising only 6.2% (Table 2). Oral speech and Vlog-style videos had higher proportions, accounting for 37.7% and 26.7%, respectively. Conversely, documentary and interview videos had lower proportions, with only 0.3% and 0.6%, respectively (Table 2). As shown in Table 2, the average MSV score was 4.49 (SD=2.23), ranging from 0 to 11. Upon closer observation, the videos exhibited the following distribution of characteristics: approximately 35% of the videos included a high level of editing (i.e. exceeding 15 cuts), around 55.8% of the videos had sound saturation, about 47.5% included background music, approximately 55.2% of the videos advocated for viewers to take action towards smoking cessation, around 33.2% of the videos had a surprise ending, and about 61.4% of the videos established message standards. The remaining elements were used in <20% of the videos. From Table 2, it can be observed that threat appeals were most commonly used (49%), followed by humor appeals (24.2%) and psychological benefit (22.1%), with economic benefit (8%) and social appeals (1.5%) being the least utilized.

**Table 2:** General characteristics of sample video clips, Bilibili 2022 (N=337)

Characteristics		Marginal mean		SD		Range	
MSV^13,22-24^		4.49		2.23		0 – 11	
Communication breadth		236.58		1377.633			
Communication recognition		79317.744		337806.209			
Communication participation		165.384		720.906			
***Message appeals*** ***^25^***	***%***	***Creators types*** ***^26^***	***%***	***Video types***	***%***	***Video length***	***%***
Social	1.5	Individuals	48.1	Oral speech	37.7	<30 s	9.8
Threat	49.0	Medical workers	10.3	Vlog	26.7	30–59 s	24.3
Humor	24.2	Corporation	6.2	Showcase	17.8	1 min – 1 min 59s	29.1
Psychological benefit	22.1	Media	35.4	Acting	7.3	2 min – 2 min 59s	13.1
Economic benefit	8.0			Mashup video	3.9	min – min 59s	13.9
				Pictorial slideshow	2.4	≥5 min	9.8

MSV: message sensation value.

From the results of the chi-squared analysis, it can be seen that individual users are more inclined to create Vlog-style videos. Media and healthcare professionals are more inclined to produce oral speech videos, while corporate users are more inclined to create showcase-style videos (p<0.001) (Table 3).

**Table 3:** Number of video types according to creator types, Bilibili 2022 (N=337)

*Video types*	*Creator types*				*Total*	*χ²*	*p*
	*Media*	*Individuals*	*Medical workers*	*Corporation*			
Oral speech	87	22	18	0	127	234.06	<0.001
Vlog	3	85	1	1	90		
Pictorial slideshow	0	6	1	1	8		
Acting	4	18	0	3	25		
Mashup video	1	8	0	4	13		
Showcase	23	19	11	7	60		
News	2	0	3	1	6		
Animation	0	3	1	1	5		
Interview	0	1	0	1	2		
Documentary	0	0	0	1	1		
Total	120	162	35	20	337		

The chi-squared test revealed significant differences in the utilization of threat appeal (p<0.001), humor appeal (p<0.001), and psychological benefit (p<0.05) among different types of videos. Specifically, oral speech videos tended to employ threat appeal and psychological benefit, while acting videos were more likely to use humor appeal.

One-way analysis of variance (ANOVA) revealed significant differences in MSV levels among different types of smoking cessation videos. However, due to sample sizes of ≤2 for some video types (e.g. only one documentary video), the requirements for *post hoc* tests were not met in this study. Therefore, *post hoc* tests were not conducted on the study sample. Specifically, most video types, such as animations, news, slideshow-style videos, and mashup videos, tended to include music. Vlogs and showcase videos generally had higher levels of sound saturation. Performance-based, mashup-style, and interview-style videos often featured surprise/twist endings. On the other hand, oral speech and documentary videos typically incorporate the ‘evidence’ element.

### The communication effect of smoking cessation topic videos and its influencing factors

To examine the communication effect of smoking cessation videos, we conducted a linear regression analysis with independent variables selected from the perspectives of video creation, MSV, and message appeal. The results of linear regression analysis for dependent variables such as overall communication effect, communication breadth, communication recognition, and communication participation indicated that MSV positively influenced the communication effect of the videos (p<0.001). When the videos included message appeal elements such as threat appeals, humor appeals, and economic benefits, the overall communication effect, communication breadth, recognition, and participation of the videos were enhanced (p<0.001) (Table 4). Additionally, the overall communication effect and communication recognition were influenced by the type of video creator (p<0.001), while the communication breadth was influenced by the type of video (p<0.001) (Table 5). Further details of the linear regression analysis can be found in the Supplementary file.

**Table 4:** The frequencies of message appeal used in different video types, Bilibili 2022 (N=337)

*Message appeal*	*Video types*										*Total*	*χ²*	*p*
	*Oral speech*	*Vlog*	*Pictorial slideshow*	*Acting*	*Mashup video*	*Showcase*	*News*	*Animation*	*Interview*	*Documentary*			
**Total**	127	90	8	25	13	60	6	5	2	1	337		
**Social**												6.486	0.690
None	126	89	8	25	13	57	6	5	2	1	332		
Yes	1	1	0	0	0	3	0	0	0	0	5		
**Threat**												42.769	<0.001
None	59	57	4	19	10	13	3	0	1	0	166		
Yes	68	33	4	6	3	47	3	5	1	1	171		
**Humor**												109.329	<0.001
None	115	82	6	3	3	38	4	3	1	1	256		
Yes	12	8	2	22	10	22	2	2	1	0	81		
**Psychological benefit**												20.494	0.015
None	91	74	7	25	13	43	6	3	1	1	264		
Yes	36	16	1	0	0	17	0	2	1	0	73		
**Economic benefit**												15.814	0.071
None	117	86	6	25	13	50	6	5	2	1	311		
Yes	10	4	2	0	0	10	0	0	0	0	26		

**Table 5:** The differences in message sensation value among different types of videos, Bilibili 2022 (N=337)

*Video types*	*Total*	*Message sensation value*		*F*	*p*
		*Marginal mean*	*SD*		
Oral speech	127	2.843	1.359	39.575	<0.001
Vlog	90	4.256	1.458		
Pictorial slideshow	8	5.250	0.886		
Acting	25	6.960	1.695		
Mashup video	13	8.000	1.354		
Showcase	60	6.233	1.899		
News	6	4.833	2.714		
Animation	5	6.000	2.550		
Interview	2	6.500	2.121		
Documentary	1	5.000	0.000		
Total	337	4.496	2.228		

SD: standard deviation.

## DISCUSSION

This study conducted a content analysis of smoking cessation videos on Bilibili and investigated factors influencing the communication effect of these videos. Based on social cognitive theory, individuals exposed to videos may learn about smoking and its consequences, which can influence their smoking behavior. Although it is challenging to measure the impact of watching videos on smoking behavior, viewers’ reactions to the videos can be used to evaluate the effectiveness of smoking cessation videos on a promotional and educational level. Understanding the factors that influence the communication effect of videos is crucial for promoting smoking cessation on social media platforms, particularly video platforms. The findings of this study indicate that MSV and message appeal elements such as threat appeals, humor appeals, and economic benefits significantly influence the communication effect of smoking cessation videos. In contrast, the impact of video types and creator types on the communication effect is limited.

### MSV and smoking cessation promotion for youth groups

This study found that MSV significantly influences the communication effect of smoking cessation videos. Higher MSV scores are associated with better communication effects of smoking cessation videos. Additionally, the variance results showed significant differences in MSV among different video types. Mashup videos had the highest average MSV score (marginal mean=8.00, SD=1.35), although they had the lowest number of videos. On the other hand, oral speech videos had the lowest average MSV score (marginal mean=8.00, SD=1.35), but they had the highest number of videos.

The study also found that viewers are more likely to watch videos with impressive content, such as powerful images, actions, and fast-paced sequences^22^. Attractive visuals can create a stronger association with smoking, making people perceive significant benefits and lower risks^29^. Research suggests repeated and frequent exposure to smoking cessation messages containing MSV elements can change people’s attitudes toward smoking or maintaining a non-smoking status^30^. This study suggests that video creators on social video platforms can incorporate more MSV elements into their video production to enhance the persuasiveness of smoking cessation-themed videos.

### Message appeal and smoking cessation promotion for youth groups

The results demonstrated that videos with message appeal elements such as threat appeals, humor appeals, and economic benefits have better communication effects^31^. In this study, 49% of smoking cessation-themed videos on Bilibili contained threat appeals. Video creators often use threat appeals to emphasize the irreversible harm caused by smoking to the body and the negative influence on individuals in the same environment. Threat appeals can change attitudes by arousing sufficient fear and enhancing people’s responses to the information^15^.

Humor appeals are also an essential factor influencing the communication effect of smoking cessation videos^18^. Humor appeals are often found in advertisement videos or narrative-based videos. When humor is combined with execution cues such as music, intriguing interludes, and charming models in advertisements or narrative videos, they evoke positive effects in the audience, such as excitement, joy, and warmth^25,32^. This study suggests that creators of smoking cessation-themed videos can consider incorporating humor elements to evoke positive emotions and responses among young audiences regarding smoking cessation.

While primary smoking prevention among youth is crucial, promoting smoking cessation directly benefits economic savings^33^. The study also found that although videos with economic benefit elements were relatively few, they still influenced the communication effect of the videos^34^. Therefore, when creating smoking cessation-themed videos, creators can consider incorporating elements related to economic benefits to emphasize the significance of smoking cessation in terms of financial savings.

### Video content and smoking cessation promotion for youth groups

Certain video types on social media platforms receive better communication effects due to public preferences^20^. Therefore, regarding smoking cessation videos on Bilibili, video types influence the communication breadth. Further analysis using one-way analysis of variance revealed significant differences in the breadth of communication among different video types (p<0.001). Therefore, video creators must select appropriate types for creating smoking cessation-themed videos^35^.

Furthermore, the study results indicated that the type of video creator particularly impacts the overall communication effect and communication recognition. Specifically, smoking cessation-themed videos created by corporate users tend to have better overall communication effects and higher recognition. Although corporate users accounted for only 6.2% of the participation in creating smoking cessation-themed videos, their substantial resources in the workforce and finances allowed for well-produced videos, resulting in better overall communication effects and video recognition. Additionally, videos created by healthcare professionals also achieved sound overall communication effects and recognition. It is likely because healthcare professionals often possess high authority in health communication on social platforms, and the public trusts their statements^36^.

### Limitations

Despite the significant findings of this study, several limitations should be considered. Firstly, this study only focused on smoking cessation videos in 2022, which may not fully represent the temporal dynamics. Secondly, the communication effects of smoking cessation videos on Bilibili only reflect the attitudes and perceptions of Chinese youth towards smoking cessation. In contrast, the communication effects of smoking cessation videos on other social media platforms warrant further exploration. Thirdly, future research should examine to what extent video effectiveness studies reflect the effectiveness of smoking cessation campaigns.

## CONCLUSIONS

This study demonstrates that social video platforms can significantly promote smoking cessation among youth, serving as essential avenues for relevant organizations and professionals to reach out to young smokers. In terms of specific video production, video creators can consider the preferences of young audiences and incorporate high MSV elements, such as visual effects, background music, and evidence, into their videos. Additionally, message appeal factors such as threat appeals, humor appeals, and economic benefits are beneficial for achieving better results in promoting smoking cessation among youth.

## Data Availability

The data supporting this research are available from the authors on reasonable request.
